# Roadblocks to Integration; Faculty’s perspective on transition from Traditional to Integrated Medical Curriculum

**DOI:** 10.12669/pjms.37.3.3217

**Published:** 2021

**Authors:** Asma Hafeez, Brekhna Jamil, Aaiz Feroze Khan

**Affiliations:** 1 Dr. Asma Hafeez, FCPS, MHPE. Anatomy Department, HITEC-Institute of Medical Sciences, Taxila, Pakistan; 2 Dr. Brekhna Jamil, MHPE. Institute of Health Professions Education & Research, Khyber Medical University, Peshawar; 3 Aaiz Feroze Khan Fazaia Medical College, Islamabad, Pakistan

**Keywords:** Curriculum, Undergraduate, Faculty, Medical, Medical institution

## Abstract

**Objective:**

This study was conducted to explore the faculty’s opinion regarding factors impeding practical transition from traditional to integrated medical curriculum at the outset and a few years after the process.

**Methods:**

This qualitative exploratory study was conducted from April 2018 to October 2018 at two undergraduate medical colleges; one where integrated curriculum was at the outset and the second running it successfully. A total of 12 semi-structured interviews (six from each college) were recorded and transcribed. Thematic content analysis was carried out and faculty’s perceptions about factors impeding practical transition to integrated curriculum were explored at two stages, i.e., at the outset and after its implementation.

**Results:**

Four impediments identified at the outset were deemed genuine by faculty who had gone through the experience including, faculty’s resistance, lack of training, lack of incentives, and insufficient resources. Four more impediments were identified after the experience including lack of leadership, lack of attention to faculty’s concerns, lack of communication and difficulties in setting appropriate assessment.

**Conclusions:**

Several factors if ignored can result in failure of integration of curriculum in undergraduate medical colleges. Relevantly appropriate policies should be outlined by the regulatory body to ensure the control on the impediments.

## INTRODUCTION

The face of the medical curriculum is constantly evolving in quest of a doctor who is trained for providing contextually appropriate services - a charge entrusted upon medical colleges; determined by the curriculum they teach.^1^ Since the Flexner report, the predominant curriculum has remained traditional,^2^ which, however, is reported causing segregation between theory and practice, and consequently demotivating students.^3^ The failure of the traditional models to meet the current needs and inter-disciplinary inquiry demands a solution^4^ which is frequently regarded to be, the globally popular, and now included in the licensing standards of numerous accrediting bodies, the Integrated curriculum.^2^,^5^

The switch towards an integrated curriculum has been attempted repeatedly over a few decades with nothing meaningful to show for it, save small incremental changes, resulting in an exhaustive cycle of “change without difference” which urges further exploration to devise a strategy that could break the trend of “recommending but not effecting integration”.^5^

While forwarding plans for curricular reforms, faculty, the tool through which curricula are developed and delivered, must be considered^4^ since their involvement and in pour of opinions promotes accountability and upgrades course quality.^6^

The current status of medical curriculum of Pakistan needs to be tailored to the local context, since such a curriculum is considered the most suitable one.^7^ Literature addressing students’ perceptions towards the integrated curriculum far outweighs that of the faculty’s perceptions towards the same,^8^ with just one recent study from Pakistan reporting faculty’s perceptions towards integrated curriculum before its implementation and identifying both promoting and impeding factors.^1^

No study could be found identifying the perceptions of faculty about the impediments faced in transition from traditional to integrated curriculum, both at the outset and post-implementation in Pakistan, representing a process of evolution with time. This study was designed to explore the faculty’s perceptions about factors impeding the practical transition from traditional to integrated medical curriculum from the outset, to a few years after transition.

## METHODS

This was a qualitative exploratory study, conducted from April 2018 to October 2018 at two undergraduate private sector medical colleges to explore the faculty’s opinions regarding factors impeding practical transition from traditional to integrated medical curriculum. The medical colleges belonged to two categories: College-A where transition to integrated curriculum was at the outset and College-B where integrated curriculum was running for nine years. Ethical approval was granted from ethical board of Khyber Medical University, Peshawar and also from ethical boards of both colleges. Participants from both colleges were selected based on purposive sampling, ensuring maximum variation. Following consultation with heads of Medical Education Department of both colleges, a mixed pool was created with faculty from senior to junior and from basic and clinical sciences departments without any gender discrimination.

After taking informed consent, the faculty members from the mixed pool of maximum variation were approached for semi-structured interviews after informed consent. Theoretical saturation was reached after a total of twelve semi-structured interviews (six from each college) and were fictitiously numbered A1 to A6 for College A and B1 to B6 for College B. This sample size was not decided beforehand, as doing it *a priori* has certain inherent problems, and saturation should be considered important in determining sample size *posteriori*, in qualitative research.^9^ All interviews were conducted one by one by the principal researcher. Following two questions with eight sub-questions, aligned with the objectives were enlisted and validated by five expert medical educationists:

### Question-1:

What are your perceptions regarding factors that impede the practical transition from traditional to integrated curriculum? (from both College-A and B)

### Question-2:

Can you identify the factors that impede successful implementation and continuation of integrated curriculum in your experience? (only from College-B)

Data collection and analysis were conducted side by side. The interviews were transcribed and thematic content analysis was carried out following the Braun’s and Clarks framework.^10^ The following sequence was employed: familiarization with data by reading the transcripts repeatedly, generation of initial-codes by the open code technique, grouping of codes into categories/axial-codes and identification of final themes after review. Throughout the process, the codes and themes were shared among principal researcher and assistant researcher; all disparities were addressed till a bilateral consensus was achieved through an iterative process.

For quality assurance; the audios and transcripts are kept safe, triangulation^11^ across data resources (by engaging a range of participants), across researchers (principal and assistant) and across sites (two colleges) was adopted, transcripts were shared with participants for member checking,^12^ thick and rich descriptions were recoded, and an audit trail was maintained with an external medical educationist.^11^ The researcher’s reflexivity was employed by “Bracketing”, and keeping reflective memos.^13^

## RESULTS

Data from faculty of two different colleges was collected. A total of twelve semi-structures interviews (six from each college) were conducted.

Question-1 was directed at exploring faculty’s perceptions about the impediments to transition to integrated curriculum. The respondents from College-A being at the outset of the process expressed the factors which they were currently experiencing, while those from College-B expressed what they felt and experienced before and during the process. The transcripts were analyzed. Thematic content analysis of the transcripts was done by the following process.

The initial-codes were grouped into axial-codes. Based on axial-codes, six final themes as impediments were derived including faculty’s resistance, faculty’s insecurity, lack of training and sensitization, lack of incentive, insufficient resources and mis-aligned curriculum. (Table-I). A few verbatim quotes representing initial codes can also be seen in Table-I.

**Table-I:** Initial-codes, axial-codes, final themes and a few representative quotes (Question-1, from both colleges).

Initial-Codes	Axial Codes	Final Themes	Representative Quotes
Why to change			“Why to change? Is there any need?” (A-5) “Integrated curriculum is not faculty’s comfort zone, while traditional is. So, they resist to leave it” (A-6) “In the beginning it did not make sense to us… there was resistance.” (B-3) “Senior faculty is the least bothered person” (B-4)
Doesn’t make sense	Resistance of faculty		
Not the comfort zone		Faculty’s resistance	
Senior faculty not bothered	Attitude of senior faculty		
Senior faculty resists most			
Fear to change			“It’s important that I don’t lose control on my subject.” (A-5) “Subjects not included in assessments are given time at the cost of the subjects which are” (A-5) “There was fear to change.” (B-3)
Faculty is threatened	Insecurity of faculty	Faculty’s insecurity	
My subject is threatened	Apprehension about control on subject		
Loss of control			
Lack of training	Lack of training		“If there is lack of training of faculty then there will be difficulty in transition.” (A-4) “Faculty would work more, give them some incentives please” (A-6) “It is called integrated system but we don’t have its basic knowledge” (A-5)
Don’t know much	Lack of awareness about integrated curriculum	Lack of training and sensitization	
What is integrated curriculum			
Lack of incentive	Lack of incentives	Lack of incentives	
Less staff	Insufficient human resources		“Proper documented guideline of basic framework is missing.” (A-5)
Less faculty			
Insufficient infrastructure			“Insufficient logistics was an impediment. There was lack of resources, we needed more faculty members, more physical space, IT support for modern methods of teaching…I think more money was required…” (B-2)
Insufficient technical support	Insufficient logistic support		
Insufficient equipment		Insufficient resources	
No budget allocation	Insufficient finances		
Need more funds			
No documented guidelines	Insufficient documentation		
No proper definitions			
Undue slashing of hours			“In integrated curriculum the teaching slots are shared among departments, subjects suffer due to incoherence between length of course and allotted time slots. Moreover, the curricular framework is inappropriate.” (A-6) “Our subject is being slashed down.” (A-5)
Complex topics need more time			
Inappropriate curricular framework	Mis-aligned curriculum	Mis-aligned curriculum	
Undue slashing of subject			

The Question-2 was asked from faculty representing College-B only, to explore their perceptions after being through the transition, hence in a position to comment on the factors affecting its sustainability. They were asked about the earlier identified impediments being genuine or not, and any further impediments which they could have identified post-experience.

Out of six identified themes from Question-1, those of faculty’s resistance, lack of training, lack of incentive and insufficient resources was deemed genuine by the participants; however, they opined that faculty’s insecurity and lack of sensitization frizzled with passage of time after repeated evaluation and modifications in curriculum. A mixed response was generated about mis-aligned curriculum, though majority considered it non-genuine, a senior faculty member thought otherwise.

Four additional impediments were identified by the faculty of College-B, post-experience including; lack of leadership, lack of attention to faculty’s concerns, lack of communication and difficulty in setting appropriate assessment.

## DISCUSSION

Three categories of impediments were identified; the ones identified at the outset, the ones deemed genuine after experience, and the ones identified after experience. The former two categories are likely to have an effect on smooth transition, while the later on sustainability of program.

Change is a difficult phenomenon in any setting generally and academic settings specifically.^14^ The faculty’s resistance to change and its attitude has been reported as impediments,^3^,^8^ and could be attributed to age and competing agendas,^1^ failure of leadership to provide suitable turf,^8^ lack of appropriate competencies due to deficiency of relevant training,^14^,^15^ time, incentive^8^,^14^ or even fear of losing professional identity. The underlying phenomena must be explored and addressed if change is desired in any educational program.^14^

In the present study, resistance of faculty was found to be genuine concern, and if not handled properly, it could pose detrimental effects on curricular reform, as has even been implicated for failure of effective running of the desired curriculum at the University of Trondheim, Norway.^16^

The theme of faculty’s insecurity was based on codes derived from College-B only. Main basis of which was concern of faculty regarding loss of subject content. The similar concerns were found earlier while integrating physiology curriculum,^17^ however, we found it to be not-genuine as these feelings go away once the appropriate modifications are incorporated after regular re-evaluations, as is recommended for any successful academic program.^18^

Lack of training was considered a genuine impediment after experience by College-B faculty, however, when asked about lack of sensitization felt earlier, the participants opined that with gradual process of transition and appropriate training, it fades away.

The faculty needs support through familiarization while embarking on a curricular reform.^19^ Ignoring ongoing training of faculty may disrupt the progress.^20^ Lack of training also leads to faculty’s resistance.^14^,^15^ Therefore, appropriately planned training can resolve the bigger impediment of resistance, as has been reported in literature,^21^ and by the participants from College-B.

Developing and running a curriculum is a very tedious job.^18^ Faculty already burdened with several assignments, finds it hard to stay enthusiastic about curriculum. The impact of research on career growth triggers faculty’s involvement, while lessens its desire to invest time in curriculum adding to resistance.^22^ Similar to our results, lack of incentive has been reported as an impediment previously as well.^1^,^8^,^21^ Providing incentives might help in dissipating the faculty’s resistance as well.

Running the integrated curriculum is a resource-intensive job, hence, lack of resources was, unsurprisingly, identified as impediment by both colleges. While College-A faculty was concerned about insufficient documentation, College-B faculty was worried about lack of technical support and finances. It was also considered a genuine impediment post-experience; hence non-provision of adequate resources can affect the smooth and sustainable transition to integrated curriculum. This is in accordance with already published fact that limited resources put a limit on the type and number of achievable objectives.^18^

The theme of misaligned curriculum emerged from codes of incoherent length of course and time slots, inappropriate curricular framework and undue slashing of subject. The similar concerns were documented while integrating a Physiology curriculum.^17^ When probed about its authenticity, only one senior faculty member from College-B found this impediment to be genuine being dissatisfied with the curricular framework, otherwise a predominantly negative response was observed. The dissatisfaction by that faculty member might be attributed to resistance to change. The majority however stated that now their subject is being given appropriate coverage and time based on regular feedback, evaluations and modifications. This is not unexpected in light of the fact that regular evaluations and requisite modifications are important for running integrated curriculum sustainably.^19^

Four additional impediments identified by College-B after 9 years of transitioning can be safely assumed to affect the sustainability of program. The lack of an all-embracing leadership is detrimental for any curricular reform. An effective leadership can achieve a successful curricular transition even in resistant institute.^23^ It depends on leaders to foresee, identify and address the barriers to make desired curricular changes effective. It is not surprising that lack of leadership is seen as an impediment for smooth and sustainable transition to integrated curriculum by College-B participants. They must have experienced the important roles leaders can play, because if leadership is willing and effective, other impediments can also be dealt with.^24^

Frequently, the goals of, and methods adopted for evaluation are poorly reported, comprehended and utilized. This limits the sustainability and positive growth of integrated curriculum.^2^ Faculty is an important stakeholder in curricular development and execution; hence any attempt to transform a curriculum should begin with bringing teachers on board and keeping them in the center.^5^ The complexity of educational change is frequently underestimated by giving primary focus to structural, external and technical elements and ignoring peoples’ emotions regarding that change process. As it involves quite many human elements, a failure to achieve the desired goals can be encountered by ignoring the emotional elements or giving them minimalistic value.^25^

These evidences endorse the impediment identified by our participants as one of them said, “You make the principles and you make the policies and then we implement them. So, our part of the story should be heard, what problems we face, because I think only then you can amend your policies” (B-3)

Teaching the traditional curriculum in isolation hardens the boundaries between different disciplines. The turf contaminated by traditional curriculum makes communication across disciplines a redundant idea and fosters resistance among faculty. On the other hand, the major themes around which an integrated curriculum revolves include inter-disciplinary teaching, building curricular links and sequencing curricular contents. All these depend upon inter-disciplinary communication.^3^

The theme of lack of communication identified as an impediment is in accordance with the fact that successful integration of basic and clinical knowledge needs the interdisciplinary boundaries to fade,^2^,^26^ and a proper plan for collaboration by formulating multi-level committees should be adopted.^3^,^18^

College-B faculty discoursed that better selection of assessment tools is needed; otherwise, students tend to opt for selective studies. Assessment not only derives learning but also is its facilitator.^27^ Selection of assessment methods is an important step in integrating the curriculum. The success of integrated curriculum is possible only when integrated assessment suitable for checking desired depth of knowledge aligned with teaching and learning methods and objectives is applied.^19^

A question is frequently pondered about failure of integration of medical curriculum to get desired outcomes.^5^ One of the factors might be hidden in mis-aligned assessment, as evidence points that faculty is generally not appropriately equipped with effective evaluation and feedback skills across different competencies.^28^

This impediment was not realized at the outset but its importance was realized along the way. The faculty needs training for developing well-aligned assessment across disciplines and competencies.^28^ Ignoring this and failure in setting integrated assessment is an effective formula for failure of integrated curriculum.^19^

### Limitations:

Due to time-constraint, the study focused only on faculty and could not be conducted prospectively, where same faculty could have opined pre and post experience, which if conducted in future, can provide true depiction of evolution overtime. Furthermore, triangulation with students’ perceptions in future can add to validity of the results.

## CONCLUSION

This study has identified several impediments that can affect the smooth and sustainable transition to integrated medical curriculum. For successful transition, such policies should be laid down which ensure curbing of these impediments and consequently ensuring faculty of their involvement in the curricular reform.

### Authors’ Contribution:

**AH:** Conceived designed data analysis manuscript editing.

**BJ:** Review final approval of manuscript and accountable for the accuracy or integrity of the study.

**AFK:** Manuscript writing data analysis.
